# Influenza-Associated Ocular Complications: A Comprehensive Review of Viral Subtypes, Clinical Presentations, and Vaccination Risks

**DOI:** 10.3390/vaccines13090950

**Published:** 2025-09-05

**Authors:** Yuan Zong, Shuang Qiu, Jing Zhang, Mingming Yang, Yaru Zou, Jingheng Du, Kyoko Ohno-Matsui, Koju Kamoi

**Affiliations:** 1 Department of Ophthalmology, Zhongshan Torch Development Zone People’s Hospital, Zhongshan 528436, China; zongyuan666@gmail.com (Y.Z.); 18925301120@163.com (S.Q.); 2 Department of Ophthalmology & Visual Science, Graduate School of Medical and Dental Sciences, Institute of Science Tokyo, Tokyo 113-8510, Japan; zhangj.c@foxmail.com (J.Z.); yangmm-12@outlook.com (M.Y.); alicezouyaru519@gmail.com (Y.Z.); k.ohno.oph@tmd.ac.jp (K.O.-M.); 3 International Ocular Surface Research Center, Institute of Ophthalmology, and Key Laboratory for Regenerative Medicine, Jinan University Medical School, Guangzhou 510632, China; dxy3662198@stu2022.jnu.edu.cn; 4 Department of Ophthalmology, The First Affiliated Hospital of Jinan University, Guangzhou 510630, China

**Keywords:** influenza virus, ocular complications, vaccination safety, H7 subtype, pathogenic mechanisms

## Abstract

This comprehensive review examines the multifaceted interactions between influenza viruses and the ocular system, integrating viral pathogenesis, clinical manifestations, and vaccine-related considerations. Influenza A subtypes (H7, H1N1, H5N1) and influenza B viruses induce a spectrum of ocular complications, from mild conjunctivitis—predominantly associated with H7 avian strains—to sight-threatening disorders like uveal effusion syndrome, acute macular neuroretinopathy, and optic neuritis. Experimental evidence confirms viral replication in human corneal and retinal cells, with H7N7 demonstrating unique tropism for ocular tissues via NF-κB-mediated inflammatory pathways. Clinical cases highlight direct viral invasion and immune-mediated mechanisms, such as Vogt–Koyanagi–Harada disease exacerbation and retinal vasculitis. Rarely, influenza vaccination has been linked to oculorespiratory syndrome, uveitis, and demyelinating events, though large-scale epidemiological studies (e.g., WHO safety reports) confirm vaccines’ favorable risk–benefit profile, distinguishing true adverse events from temporal associations. This synthesis emphasizes the need for ophthalmologists to prioritize surveillance during influenza seasons, integrating diagnostic tools like conjunctival RT-PCR and optical coherence tomography. Future research should focus on defining viral receptor-binding mechanisms in ocular tissues and developing targeted therapies for severe retinopathies, while reinforcing vaccination as a cornerstone of public health despite rare ocular risks.

## 1. Introduction

According to the World Health Organization, influenza epidemics affect up to 20% of the global population each year and result in between 290,000 and 650,000 deaths worldwide [1,2]. The Orthomyxoviridae family comprises four genera of influenza viruses: influenza A, B, C, and D [1,3]. Among these, influenza A viruses (IAVs) are particularly significant because of their potential to cause severe pandemics marked by high morbidity and mortality rates [3,4].

The influenza viruses that cause respiratory infections also have substantial effects on human eye health. The virus family affects different parts of the eye, ranging from the anterior to the posterior segments [5]. In addition to virus-induced complications, the literature also shows that influenza vaccinations can lead to various ocular complications [6,7]. Post-vaccination complications include mild to moderate conjunctivitis alongside severe conditions such as panuveitis, exudative retinal detachment, and optic neuritis [5,6,7,8,9,10,11].

Given the multifaceted impact of influenza viruses on ocular health and the potential risks associated with vaccination, ophthalmologists need to be vigilant and fully understand these possible complications. Therefore, this review presents a detailed overview of the effects of influenza virus infections on the eyes by examining symptoms associated with various viral subtypes, their pathologic processes, and vaccine-related ocular complications. The research aims to compile a comprehensive clinical guide for identifying and managing ocular complications associated with influenza/influenza vaccination through a thorough review of published studies, as well as to suggest avenues for future research.

## 2. Methods

This review systematically analyzed influenza virus-related ocular complications and vaccine-associated adverse events. A literature search was conducted in PubMed, Web of Science, and Embase through May 2025 using keywords like “influenza virus”, “ocular manifestations”, “H7N7”, and “influenza vaccine adverse events”. Eligible English studies included clinical trials, case series, and reviews on viral ocular tropism, clinical symptoms, or vaccine safety. Data extraction covered study characteristics, viral subtypes, ocular findings, and outcomes. Emerging evidence on H7 subtypes and recent vaccine safety data were incorporated from peer-reviewed articles and conference proceedings. The review synthesizes findings by pathogenesis, clinical manifestations, and vaccine implications.

## 3. Basic Characteristics and Epidemiology of Influenza Viruses

Influenza virus, a respiratory pathogen of significant public health importance, belongs to the Orthomyxoviridae family. It is characterized as an enveloped virus containing segmented, single-stranded negative-sense RNA and is classified into four types (A–D), with distinct structural and life cycle features critically influencing pathogenicity and transmissibility [4,12,13,14]. Table 1 systematically compares the clinical and epidemiological characteristics of the four influenza types, highlighting their distinct host ranges, clinical manifestations, and transmission patterns.

Several important influenza A pandemics have occurred after following seasonal patterns throughout human history. The most devastating was the 1918–1919 “Spanish Flu” pandemic, caused by the A (H1N1) virus, which infected approximately one-third of the world’s population (around 500 million people) and resulted in 20–50 million deaths [2,15]. The 1957–1958 “Asian Flu” pandemic, caused by the A (H2N2) virus, originated in Guizhou, China, and resulted in 1–4 million deaths globally. The virus was a reassortment between avian and human influenza strains [16,17].

The 1968–1969 “Hong Kong Flu” pandemic, caused by the A (H3N2) virus (descended from the 1957 H2N2 virus), also claimed 1–4 million lives. The H3N2 strain, which emerged in 1968, continues to spread as a seasonal influenza virus in current times [18,19]. The 2009–2010 pandemic was caused by the A (H1N1) pdm09 virus. The strain emerged from a genetic combination of pig and bird and human elements which spread across 214 countries and resulted in 284,000 fatalities [2,20]. It is worth emphasizing that, although commonly referred to as “swine flu”, the pathogen is a novel strain of the A (H1N1) virus that was derived from several virus lineages, including the North American H3N2 triple-reassortant, the classical swine H1N1 lineage, and the Eurasian ‘avian-like’ swine H1N1 virus [21]. Now, H1N1 and H3N2 continue to circulate globally as predominant seasonal influenza strains, with A (H1N1) pdm09 having replaced the previous seasonal H1N1 following the 2009 pandemic [22,23].

Ophthalmologists should pay attention to H7-subtype IAVs (H7 Avian influenza) because of their importance. The IAV H7N9 strain continued to be reported in China and the United States in 2017 [24,25], and reports show H7-subtype IAVs caused conjunctivitis in human patients with H7N7, H7N3, and H7N2 infections [26,27,28,29,30,31,32,33]. In some case reports, positive results for the influenza virus were also detected in eye swabs [26,29,31,33,34].

## 4. General Symptoms and Subtype Differences in Influenza Virus Infection

Respiratory symptoms, such as dry cough, sore throat, nasal congestion, and rhinorrhea, often accompany systemic manifestations. Patients may experience sudden-onset fever (body temperature > 38 °C), chills, headache, generalized muscle and joint aches, as well as fatigue, along with other systemic manifestations. Most patients will recover spontaneously within 1–2 weeks; however, some individuals may progress to severe diseases. Common complications include pneumonia (viral or bacterial), bronchitis, sinusitis, otitis media, and exacerbation of pre-existing chronic conditions. Elderly individuals (aged ≥65 years), young children (<5 years old), those with immunodeficiency, pregnant women, postpartum women within 2 weeks of delivery, people living in group settings, and those with chronic underlying conditions or obesity are more prone to developing complications [35,36,37]. While these general clinical features are commonly observed, each influenza type demonstrates distinct characteristics in terms of severity, host specificity, and epidemiological patterns, as detailed in Table 1.

Regarding IAV, its infection can induce typical influenza-like symptoms, including fever, cough, headache, etc. The severity of the illness can vary from mild to severe; critically ill patients may present with respiratory failure, acute respiratory distress syndrome, septic shock, etc. [2,38]. Notably, septic shock may arise either as a direct consequence of viral infection (mediated by virus-induced cytokine storm and endothelial injury) or as a complication of secondary bacterial infections in critically ill patients. These pathogenic mechanisms frequently coexist and may exhibit synergistic effects in clinical settings [39]. Among influenza A viruses, H1N1 and H3N2 are the most predominant seasonal influenza virus subtypes. In comparison with H1N1, H3N2 often causes more severe illnesses, especially among the elderly population. Studies have demonstrated that H3N2 infections are associated with higher hospitalization rates and mortality rates and are more likely to result in complications. The antigenic drift rate of the H3N2 virus is relatively rapid, which affects vaccine efficacy and is also one of the reasons for its stronger pathogenicity [18,23,40,41].

The clinical manifestations of IBVs are similar to those of IAVs [42,43,44,45]. In children, IBV infections are more likely to cause benign acute myositis; the duration of biphasic fever may be longer than that in IAV infections [44]. Although the hospitalization rates and mortality rates of IBV and IAV are comparable in the general population, IBV may lead to more severe diseases in children [45,46].

ICV typically causes upper respiratory tract infections, particularly among preschoolers [37]. Clinical symptoms such as coughs, fevers, and malaise are usually mild. Occasionally the virus may spread to the lower respiratory tract, leading to symptoms such as bronchitis and pneumonia, especially among infants [47,48].

IDV only causes mild symptoms such as rhinitis and tracheitis in experimental infections; viral replication is usually confined to the upper respiratory tract [37,49].

## 5. Infection Mechanisms, Pathogenesis and Experimental Evidence of Influenza Virus Interactions with Ocular Tissues

The interaction between influenza viruses and ocular tissues involves intricate virus–receptor interactions and host immune responses. Initially, the human ocular surface epithelium, as a mucosal surface, is equally exposed to influenza virus-containing aerosols or contaminants as the respiratory tract [50]. Moreover, the eye and respiratory system are directly connected through the nasolacrimal duct system, facilitating bidirectional exchange of virus-containing fluids [51]. The nasal-associated lymphoid tissue further links with the ocular mucosal immune system via the nasolacrimal duct, reinforcing their immunological interdependence [52].

Human respiratory and ocular tissues ubiquitously express epithelial glycoproteins bearing terminal sialic acids (SA) [53]. The influenza viral hemagglutinin (HA) protein possesses a conserved receptor-binding site that interacts with sialic acid residues. This binding constitutes the initial step of viral infection, rendering tissue tropism of influenza viruses intrinsically linked to the tissue-specific distribution of sialic acids [54]. In humans, α2-6-linked sialic acids predominate in nasal mucosa and trachea, whereas α2-3-linked sialic acids are more abundant in lower respiratory tract tissues and ocular structures. Notably, epithelial cells in the human lacrimal sac and nasolacrimal duct co-express both sialic acid types; α2-6-linked sialic acids are detected on both epithelial and goblet cells of the lacrimal duct, while α2-3-linked sialic acids are exclusively present in epithelial cells [50,53].

This distribution pattern partially explains the tropism exhibited by certain influenza virus subtypes, particularly avian influenza viruses that preferentially bind α2-3-linked sialic acids and are associated with ocular manifestations in humans. Among these, H7 influenza viruses demonstrate the most pronounced ocular tropism, with conjunctivitis occurring in over 80% of human H7 infection cases [50,53,55]. This ocular tissue preference may be attributed to the efficient binding of H7 viruses to α2-3-linked sialic acids expressed on corneal and conjunctival epithelial cells [56].

Viral mutations have also led to alterations in the ocular tropism of influenza viruses. Early A (H1N1) isolates exhibited nearly exclusive specificity for SAα2,6 receptors, resulting in infections predominantly localized to the nasal mucosa and trachea, with rare ocular involvement [57]. However, studies on the A (H1N1) pdm09 virus have found that its HA gene is derived from the classical swine H1N1 lineage, endowing it with the ability to bind both SAα2,6 and SAα2,3 [53,58]. This adaptive change has expanded the virus’s ability to infect the lower respiratory tract tissues and ocular tissues.

Multiple laboratory studies have demonstrated that specific IAV subtypes show tropism toward the eye [56,59]. Michaelis et al. investigated IAV cytopathogenic effects and apoptosis-inducing properties through H1N1 and H5N1 virus strain infections of human retinal pigment epithelial (RPE) cells. The research team discovered that H5N1 virus strains produced high titers in RPE cells, which resulted in RPE cell death: H5N1 virus strains reached titers greater than 10^8^ TCID 50/mL in RPE cells, and the antiviral drug ribavirin blocked this virus-induced cell death. The replication of the H5N1 virus in RPE cells was inhibited by pretreatment with type I interferons (interferon-α and interferon-β) and a type II interferon (interferon-γ) [59].

Belser et al. demonstrated in animal model experiments that H7N3 and H7N9 subtype viruses could be detected in mouse eyes following ocular inoculation [60]. The team also confirmed that H7 viruses from Eurasian and North American lineages could be detected in mouse eyes after ocular inoculation but H7N2 virus isolated from human respiratory tracts was not detected [61]. Ferret models show that H1N1 and H3N2v (human influenza viruses) and H7N7 and H7N9 (avian influenza viruses) could lead to respiratory infections after exposure through the eyes, thus proving the eyes function as both virus replication sites and respiratory infection entry points [62]. Ocular aerosol contact enables fatal respiratory infections from viruses that lack known ocular tropism according to research [62].

The experimental data shows that influenza viruses infect multiple eye tissues while H7N7 viruses demonstrate unique replication patterns and cytokine expression profiles in these tissues potentially linked to influenza-related eye symptoms. Belser et al. examined the infection of H7, H5, and H1 subtype viruses in primary human corneal and conjunctival epithelial cells, finding that multiple virus subtypes can infect and replicate in various ocular cell types, with highly pathogenic H7N7 and H5N1 viruses producing the highest titers [56]. H7N7 virus infection exhibited certain inflammation-related characteristics, including elevated IL-1β levels in primary human corneal and conjunctival epithelial cells and significantly enhanced expression of genes related to NF-κB signal transduction following infection of human corneal epithelial cells, with this enhancement exceeding that observed with H5N1 or H1N1 virus infection [56].

## 6. Ocular Manifestations Associated with Influenza Virus Infection

Influenza viruses can induce a spectrum of ocular manifestations, ranging from mild conjunctivitis to severe vision-threatening conditions. While conjunctivitis represents the most prevalent ocular manifestation, particularly in infections caused by avian influenza H7 subtypes, clinical and laboratory investigations have documented more severe complications including uveitis, retinopathy, and neuro-ophthalmic disorders. These ocular manifestations may result from direct viral invasion of ocular tissues or immune-mediated responses triggered by the infection. This section reviews influenza-associated ocular presentations, clinical characteristics, diagnostic approaches, and therapeutic strategies based on published case reports and outbreak investigations. Table 2 delineates the comprehensive ocular manifestations documented in association with influenza virus infection. Based on the currently available literature, conjunctivitis has been identified as the predominant ocular complication associated with influenza virus across all subtypes [27,28,29,31,32,33,34,63,64,65], and is clinically diagnosed when patients present with two or more of the following symptoms: conjunctival hyperemia, epiphora, ocular pruritus, ocular pain, burning sensation, purulent discharge, and photophobia [29].

### 6.1. Influenza Virus-Associated Conjunctivitis

Taylor and Turner published a report in 1977 about avian influenza keratoconjunctivitis that developed from accidental laboratory exposure [26]. The patient developed follicular conjunctivitis with mucopurulent discharge that resolved independently during a two-week period. The keratitis exhibited unusual intraepithelial opacities that appeared one week after infection and disappeared completely over the following three weeks. The virus was confirmed to be the Dutch strain (Hav 1 Neq 1) of fowl plague through viral culture.

H7 subtype avian influenza viruses account for most cases of influenza-associated conjunctivitis, and the H7N7 subtype exhibits the strongest association with this eye condition. Webster et al. documented human conjunctivitis cases from influenza A virus strains isolated from seals in 1981 [27]. An investigator experienced severe conjunctivitis after an infected seal sneezed directly into their face and right eye. The researcher experienced severe conjunctivitis in their right eye after 40 h, and preauricular lymphadenopathy appeared at 96 h. The fourth day marked the beginning of improvement in conjunctivitis. The affected individual’s conjunctival swabs revealed high titers of influenza A virus, which laboratory tests identified as A/Seal/Mass/1/80 (H7N7). Laboratory tests for both hemagglutination inhibition and neuraminidase inhibition failed to detect antibodies against the seal virus in blood samples and tear fluid from the infected individual.

Kurtz et al. [34] documented the isolation of avian influenza virus from a woman who developed conjunctivitis. A 43-year-old female developed right-sided conjunctivitis after straw entered her eye during duck house cleaning the previous day. Scientists identified the isolated virus as IAV (A/England/268/96) which turned out to be an H7N7 subtype. The serological analysis of post-exposure blood samples showed no detectable antibodies against the 268/96 virus, similarly to the seal virus infection case.

Koopmans and colleagues documented that 349 out of 453 individuals with health complaints exhibited signs of conjunctivitis during the H7N7 outbreak in the Netherlands in 2003 [29]. The A/H7 virus was found in conjunctival samples from 78 patients (26.4%) who had isolated conjunctivitis, 5 five patients (9.4%) who had influenza-like illness and conjunctivitis, 2 patients (5.4%) with influenza-like illness, and 4 individuals (6%) with other symptoms. The study authors calculated that 4500 individuals in the Netherlands were exposed to A/H7-infected poultry during the outbreak and that conjunctivitis affected 7.8% of this population.

Two confirmed cases of the highly pathogenic avian influenza H7N3 emerged during the 2004 outbreak in British Columbia, Canada according to laboratory testing [30]. The patients experienced conjunctivitis alongside mild symptoms similar to influenza. The 57 confirmed and suspected cases of infection presented with various eye symptoms, including red eyes in 21% of patients, lacrimation in 11% of patients, ocular pruritus in 23% of patients, ocular pain in 7% of patients, burning sensation in 12% of patients, ocular discharge in 7% of patients, and photophobia in 9% of patients. Patients who contract H7 avian influenza subtypes show a lower occurrence of conjunctivitis compared to other infected patients. The research team led by Puzelli examined 185 serum samples from poultry workers during the H7N3 outbreak in Italy in 2003 and discovered that 7 samples (3.8%) showed substantial reactivity to both H7N3 and H7N1 viruses through serologic testing. One worker had clinical symptoms of conjunctivitis, while the rest reported no influenza-like illness symptoms [28]. Western blot analysis showed significant H7 HA reactivity in seven samples, which indicated that these individuals had either contracted H7 viruses or had been exposed to them. One of the seven seropositive individuals experienced conjunctivitis symptoms during the outbreak period. The six seronegative participants who had conjunctivitis did not show any evidence of H7 HA antibodies in their blood tests. The H7N2 outbreak in the United Kingdom during 2007 led to documented conjunctivitis cases which resulted in the detection of influenza A virus from swabbed eyes [31].

**Figure 1:**
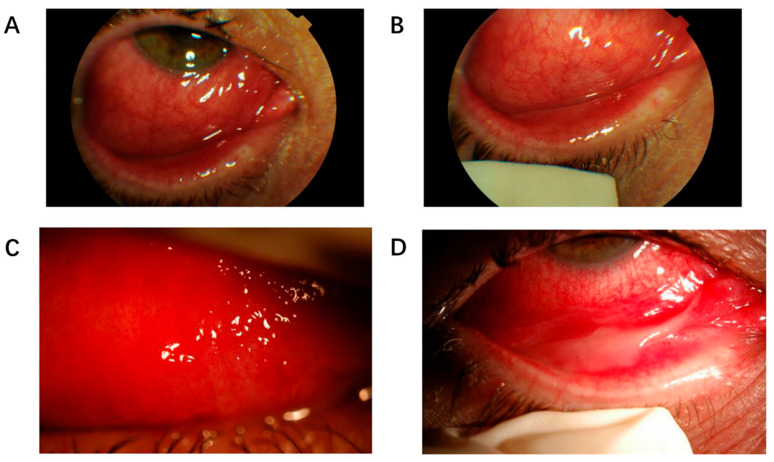
Bleeding follicular conjunctivitis induced by influenza virus H1N1. (**A**) Lower palpebral conjunctival chemosis with severe hyperemia, accompanied by significant bilateral eyelid edema. (**B**) Severe conjunctival hyperemia of the lower lid with chemosis and marked palpebral edema. (**C**) Characteristic petechial hemorrhages with prominent upper subtarsal follicular reaction. (**D**) Inflammatory pseudomembranes typical of H1N1-associated viral conjunctivitis. (Adapted with permission from Ref. [68], © 2010 Lopez-Prats et al. This work is licensed under a Creative Commons Attribution 3.0 International License, allowing reuse with proper attribution.)

Although rare, H1N1 also has been documented to induce conjunctivitis. Lopez-Prats et al. [68] documented the initial PCR-confirmed case of H1N1 viral hemorrhagic follicular conjunctivitis. A 45-year-old female patient received a throat swab diagnosis of H1N1 infection before developing unilateral acute hemorrhagic conjunctivitis five days later. The patient exhibited severe eyelid swelling along with extensive mixed conjunctival redness, moderate tissue swelling, and a pseudomembrane formation. The subconjunctival space contained multiple follicles and petechiae (Figure 1). PCR testing of secretions from the patient’s eyes showed H1N1 viral RNA only in the infected eye, while the healthy eye remained negative. The patient received symptomatic treatment for 15 days which included nonsteroidal anti-inflammatory drugs applied topically, irrigation, and topical ganciclovir administration until their symptoms improved. A young male patient also developed an erythematous rash three days after his upper respiratory symptoms and fever began, according to Koul et al.’s 2013 report about the H1N1 influenza pandemic [67]. The patient developed a subconjunctival hemorrhage in their left eye, which doctors linked to their severe coughing episodes. The patient showed positive results from receiving oseltamivir treatment.

Conjunctivitis has also been reported in association with IBV infections, as documented by Goyal in two pediatric cases of influenza B-induced rash and mucositis [64]. In the first case, bilateral conjunctival hyperemia without desquamation was observed, while the second case reported conjunctivitis without detailed specifications of its manifestation. Both patients had a history of Stevens–Johnson syndrome due to previous mycoplasma infection.

### 6.2. Uveal Disorders Caused by Influenza Viruses

In a limited number of case reports or case series, additional ocular manifestations have also been documented. Medical research shows that uveal effusion syndrome develops because of H1N1 influenza infection. An 11-year-old girl experienced severe visual loss from uveal effusion syndrome while testing positive for H1N1 influenza according to Roesel et al. [75]. The patient displayed widespread conjunctival redness along with a deep anterior chamber and cells in the vitreous and posterior segments, which indicated choroidal and subretinal exudation. The patient’s retinal/choroidal complex showed increased thickness according to ultrasonography results and fluorescein angiography showed early leakage at the posterior pole with circumscribed hypo fluorescence indicating subretinal detachment. The patient’s eyesight shifted from slight hyperopia to −6.5 diopters in a sudden manner. The combination of oral prednisolone and doxycycline with scopolamine eyedrops led to significant improvement of the condition in nine days. The exact pathophysiological process linking influenza infection to these eye complications remains unclear, although it could involve viral penetration into the eye tissue, immune reactions within the eye, or breakdown of the blood–retinal barrier during systemic immune activation.

Uveitis stands as a major eye condition which can be induced by influenza infection. An 11-year-old boy developed bilateral acute anterior uveitis and papillitis in one eye and neuroretinitis in the opposite eye after contracting an upper respiratory tract infection of influenza A virus, possibly H1N1, according to Lai et al. [71]. The patient received steroid pulse therapy, which effectively treated these conditions. A 70-year-old male developed bilateral acute anterior uveitis and unilateral optic neuritis after contracting influenza A according to Nakagawa et al. [74]. The patient experienced blurred vision and optic disc edema with left eye hemorrhage and bilateral conjunctival injection. The analysis of aqueous humor samples indicated that autoimmune mechanisms probably caused the observed ocular manifestations instead of direct viral infection. The combination of topical prednisolone acetate ophthalmic suspension with high-dose intravenous methylprednisolone produced substantial therapeutic effects. Gümbel et al. [72] reported a patient who developed bilateral panuveitis after contracting influenza A, yet research shows that eye symptoms during influenza outbreaks usually present as catarrhal conjunctivitis, with posterior segment involvement remaining exceptionally uncommon.

Infection with IAV has been linked to the development of Vogt–Koyanagi–Harada Disease (VKHD). Yoshino et al. [70] reported a 31-year-old Japanese male tested positive for influenza A virus antigens while showing VKHD characteristics through fluorescein fundus photography, with multiple diffuse early hyperfluorescent points, and optical coherence tomography revealing serous retinal detachments. The patient exhibited meningitis alongside tinnitus and mononucleosis of the cerebellar fluid. The patient received peramivir treatment for influenza A followed by methylprednisolone pulse therapy and corticosteroid administration, which led to the gradual improvement of his symptoms. The presented case indicates that influenza A virus infection could trigger or intensify the development of VKHD.

### 6.3. Retinal and Neuro-Ophthalmic Complications of Influenza

Medical studies have established that influenza infection leads to complications affecting both the retina and the nervous system. A 10-year-old male patient developed painless bilateral central vision loss one week after receiving an influenza A diagnosis according to Shields et al. [69]. Optical coherence tomography showed superficial retinal nerve fiber layer infarcts together with inner nuclear layer hyperreflectivity which indicated paracentral acute middle maculopathy and outer nuclear layer hyperreflectivity and ellipsoid zone disruption, which pointed to acute macular neuroretinopathy. The right basal ganglia showed enhancement on brain MRI, which indicated focal encephalitis. The medical team diagnosed the patient with influenza-induced leukocytoclastic vasculitis and started intravenous steroid treatment.

A 13-year-old female patient developed encephalopathy and complete blindness to light perception after 24 h of experiencing H1N1 influenza A symptoms, including fever and myalgias, according to Breker et al. [73]. Ophthalmoscopy showed widespread retinal ischemic damage and a brain MRI revealed hemorrhagic infarction of the lateral geniculate body. The patient received intravenous corticosteroids and intravenous immunoglobulin and plasmapheresis treatment but experienced no significant improvement in vision. The authors linked this case to an immunologic response to the virus that caused occlusive damage to the arteriolar endothelium, which resulted in simultaneous retinal and lateral geniculate body infarctions.

**Figure 2:**
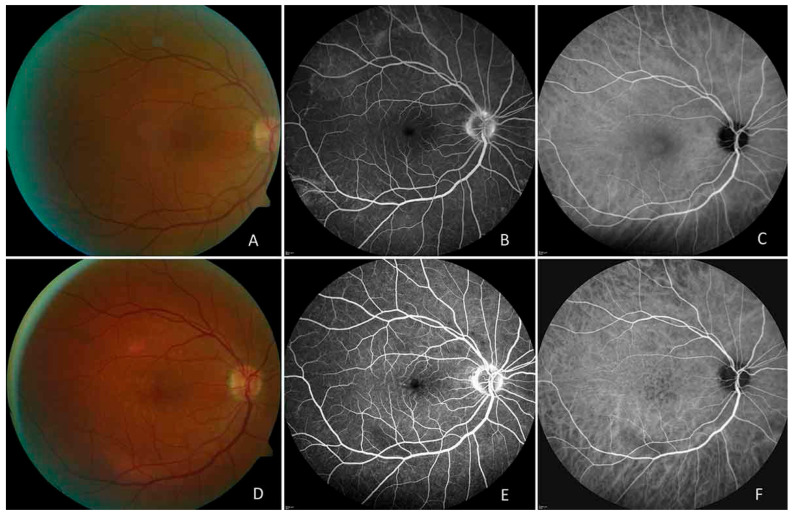
Ocular manifestations of acute posterior multifocal placoid pigment epitheliopathy (APMPPE) associated with influenza virus infection. (**A**) Color fundus photograph of the right eye demonstrating characteristic placoid lesions. (**B**) Fluorescein angiographic image showing hypofluorescence in early phase with late staining. (**C**) Indocyanine green angiographic image revealing hypofluorescent lesions with slight dye leakage at the optic disc and small foci of inflammation at the periphery before treatment. (**D**–**F**) Follow-up imaging after 6 months of systemic prednisone treatment showing significant resolution of inflammatory lesions: (**D**) color fundus photograph, (**E**) fluorescein angiography, and (**F**) indocyanine green angiography demonstrating an decreased severity of inflammation and healing of the placoid lesions. (Adapted with permission from Ref. [6], © 2025 Brydak-Godowska et al. This content is distributed under a CC BY-NC-ND 4.0 License, allowing non-commercial use only without modification and requiring clear attribution to the original work.)

Brydak-Godowska et al. [6] documented a series of cases of upper respiratory tract infection-related eye complications, which included three patients with acute posterior multifocal placoid pigment epitheliopathy (APMPPE) and one patient with serous macular detachment (Figure 2). Two patients received laboratory confirmation of influenza virus infection through molecular biological testing or hemagglutination inhibition testing. The patients received systemic prednisone treatment, which led to the resolution of active inflammatory lesions and better visual acuity.

## 7. Management of Influenza-Associated Ocular Complications

Viral conjunctivitis, including that caused by influenza viruses, typically presents as a benign, self-limiting ocular complication, with most cases resolving spontaneously within several weeks [68,76]. Supportive therapies such as cold compresses, topical antihistamines, and artificial tears can effectively alleviate local discomfort symptoms [77].

Currently, there is a paucity of specific antiviral agents targeting influenza-associated ocular manifestations. Regarding systemic treatment, the neuraminidase inhibitor oseltamivir (Tamiflu^®^) may be employed for systemic antiviral therapy in influenza-positive patients with concurrent conjunctivitis or other ocular manifestations through its mechanism of inhibiting viral replication and release [78]. Belser et al. investigated the efficacy of oseltamivir in suppressing influenza virus replication and transmission following ocular exposure by inoculating ferrets via ocular aerosol administration [79]. Their findings demonstrated that oral oseltamivir administration effectively reduced influenza viral replication in both ocular and respiratory tissues, ameliorated clinical disease manifestations, and diminished viral transmission capacity to susceptible contacts. Furthermore, in vitro experiments corroborated that oseltamivir could restrict influenza virus replication in human corneal epithelial cells [79]. The same study also demonstrated that oseltamivir effectively suppressed the replication of H7 influenza virus in mice inoculated via the ocular route [79].

Notably, prior to the 2009 H1N1 pdm09 subtype pandemic, oseltamivir resistance remained relatively uncommon; however, subsequent reports of resistant cases have increased, particularly among H1N1 pdm09-infected individuals [36]. For such oseltamivir-resistant influenza cases, intravenous zanamivir (Relenza^®^) may serve as an alternative therapeutic option [36,80].

In cases of severe ocular complications, systemic corticosteroid therapy may be cautiously considered. For instance, Bredak-Godowska et al. reported two cases of APMPPE associated with influenza infection where systemic corticosteroid administration (e.g., prednisone) led to resolution of ocular lesions and significant visual improvement [6]. As previously described, the 10-year-old male patient with IAV-associated acute macular neuroretinopathy and cerebral involvement demonstrated significant visual improvement in both eyes after 3 weeks of treatment, which included a 5-day course of intravenous methylprednisolone pulse therapy followed by a tapering regimen of oral corticosteroids [69]. At the 8-month follow-up, the patient remained asymptomatic with stable visual acuity of 20/25 in both eyes. Fundus examination revealed normal findings, while OCT showed only residual nasal retinal thinning. However, it is important to note the potential adverse effects associated with prolonged corticosteroid use.

## 8. Influenza Vaccine-Related Ocular Complications

Public health relies on influenza vaccination as its fundamental preventive measure because it decreases both the severity and death rates from seasonal influenza outbreaks. Vaccination rates still differ substantially between different populations and geographic areas, especially among vulnerable groups including elderly adults and people with compromised immune systems. The safety profile of influenza vaccines remains generally positive, but healthcare providers must monitor rare adverse events (AEs) that include ocular complications because these reports have raised concerns about vaccine safety [7]. The chapter analyzes clinical reports alongside epidemiological data to evaluate both the range of complications affecting eyes from influenza vaccines and their mechanisms and their impact on patient care and vaccine advocacy.

Oculo-respiratory syndrome (ORS) represents an adverse reaction associated with various influenza vaccines. Clinical manifestations typically comprise bilateral conjunctival erythema, respiratory symptoms (coughing, wheezing, dyspnea, or chest tightness), or facial edema, emerging within 24 h post-vaccination. Epidemiological data indicate elevated susceptibility among female recipients and individuals aged 40–59 years, with substantially increased recurrence risk (OR = 9.4–9.6) in subjects with prior ORS episodes. The risk of recurrence after revaccination was noted to be a concern, with some reluctance to revaccinate among those who previously experienced ORS [81]. The laboratory results showed that ORS patients maintained elevated levels of IL-10 and IL-3 in their plasma which indicates that individual host factors might increase the risk of developing ORS after receiving influenza vaccines [82]. Research by De Serres al. demonstrated that ORS could be linked to incomplete virus particle split in vaccines like Fluviral S/F (Shire Biologics) which resulted in a 34% vaccine-attributable recurrence rate compared to 15% with Vaxigrip (Aventis Pasteur) [83]. In terms of severity, ORS is generally considered a mild and self-limiting syndrome [81,84]. A Canadian retrospective cohort study published in 2003 involving 2070 vaccine recipients found that ORS symptoms were reported by 5.6% of Fluviral^®^ recipients and 4.8% of Vaxigrip^®^ recipients, with no significant difference between vaccines (*p* = 0.43). Among affected individuals, 42% described their experience as severe in a previous related study [81].

Multiple case studies have documented optic neuritis and demyelinating disorders following influenza vaccination [85,86,87,88]. A case–control study by DeStefano et al., however, revealed that influenza vaccination did not raise the risk of developing multiple sclerosis or optic neuritis (odds ratio 0.8, 95% confidence interval 0.6–1.2) [89]. Epidemiological surveillance data indicates a mere three cases per million vaccinations for H1N1 influenza-vaccine-associated optic neuritis [86]. When this rare complication occurs, patients typically experience rapid visual deterioration progressing to near-total blindness. However, clinical studies indicate that corticosteroid intervention (whether administered orally or intravenously) may have therapeutic effects on optic neuritis following influenza vaccination, contributing to visual improvement. The majority of patients achieve complete recovery after intervention. Delayed treatment may lead to disease progression, increasing the risk of optic nerve tissue damage and consequently impairing visual recovery [86,90].

Medical reports have documented multiple instances of uveitis occurring after influenza vaccination, which involved different symptoms. A 30-year-old Filipino male developed VKHD two days after receiving an influenza vaccination which led to bilateral pain, redness, photophobia, floaters, headache, and tinnitus, according to Murtaza et al. [91]. A 52-year-old female developed VKHD one month after receiving the influenza vaccine, according to Kim’s report [92]. Two patients developed exudative retinal detachment and uveitis after receiving H1N1 influenza vaccination, according to Tao et al. [9]. Two patients developed panuveitis with orbital inflammatory syndrome after receiving influenza vaccinations, according to Manusow et al. [9]. A study by Doukkali et al. documented bilateral posterior scleritis and panuveitis in a patient who received the 2022 Fluzone Quadrivalent influenza vaccine (Sanofi Pasteur Inc., Cambridge, MA, USA) (causal association not confirmed) [93]. A 60-year-old male developed bilateral panuveitis four days after receiving the H1N1 vaccination, according to PCR and Goldmann–Witmer coefficient results, which indicated VZV reactivation leading to acute retinal necrosis [66]. Based on the currently available case reports, systemic corticosteroid administration demonstrates therapeutic efficacy; however, prolonged disease duration may contribute to unfavorable clinical outcomes [9,91,93].

Multiple Evanescent White Dot Syndrome (MEWDS) emerged as an adverse ocular event following inactivated influenza vaccination, as documented by Goyal et al. (2013), in a patient who experienced vision deterioration, photopsias, and scotomas [8]. The patient’s symptoms resolved spontaneously within 1 month. APMPPE has been documented by Mendrinos and Baglivo [94] in a 27-year-old male who developed bilateral photophobia and metamorphopsia 14 days after influenza vaccination and by Branisteanu and Bilha [95] in an 18-year-old female who experienced painless bilateral vision decrease two weeks post-vaccination. Both cases demonstrated clinical improvement following treatment with either systemic or topical corticosteroid therapy.

There have been reported cases of acute macular neuroretinopathy (AMN), a rare retinal disorder, associated with influenza vaccination. A 42-year-old female developed AMN and black spots and paracentral scotoma after receiving seasonal influenza vaccination according to Shah et al. [10]. De Salvo et al. reported a case series of six AMN patients, including one case potentially associated with influenza vaccination [96]. In both reports, the patients demonstrated AMN improvement through conservative management over several months [10,96]. Given the limited number of reported cases, the potential causal relationship between AMN and influenza vaccination remains undetermined.

The medical literature documents two cases of corneal graft rejection after influenza vaccination: Solomon and Frucht-Pery [11] reported bilateral simultaneous rejection while Hamilton et al. documented deep anterior lamellar keratoplasty rejection [97]. In addition, Ho et al. recently documented bilateral cystoid macular edema and corneal endothelial graft rejection after patients received influenza and varicella zoster vaccinations [98]. These findings suggest a rare yet clinically significant association between corneal graft rejection and influenza vaccination. Although the incidence of this complication is exceedingly low, it may lead to severe visual impairment or even blindness. Notably, the corneal graft transparency in the aforementioned cases demonstrated improvement following corticosteroid therapy [11,97,98], indicating that prompt intervention may mitigate the severity of rejection episodes. Nevertheless, current vaccination strategies for corneal transplant recipients lack systematic guidelines, and clinical decision-making remains uncertain regarding several critical issues: whether influenza vaccination should be routinely recommended for corneal transplant patients, whether immunosuppressive regimens should be adjusted peri-vaccination, and whether additional anti-rejection prophylaxis should be implemented.

Moreover, instances of exceptionally rare ocular complications associated with influenza vaccination have also been documented in the medical literature. A 25-year-old male developed unilateral acute idiopathic maculopathy eight days following his H1N1 vaccination (Sanofi S.A.), according to Jorge et al. [99]. A 2-year-old boy developed unsteady gait four days after his first inactivated H1N1 influenza vaccination (Arepanrix) before experiencing an inward-turning left eye following his second dose and eventually developed bilateral optic disc swelling with venous engorgement, according to Lapphra et al. [100].

The findings from large-scale safety studies help clarify the reported cases. Hashimoto et al. performed a self-controlled case series analysis to evaluate influenza vaccine-related eye complications in 4527 Japanese adults who were 65 years or older [101]. The study results indicated that the incidence rate ratio for uveitis, scleritis, retinal vein occlusion, retinal artery occlusion, and optic neuritis remained at 0.99 within 56 days post-vaccination compared to control periods (95% confidence interval 0.87–1.14). This extensive evaluation reduces worries about eye-related side effects that older adults experience after receiving influenza vaccinations.

The study by Skowronski et al. revealed that children with diabetes and their siblings experienced ORS at a rate of 13% (95% confidence interval 10–16%), with respiratory symptoms that typically resolved within two days for 72% of cases [102].

## 9. Conclusions

This comprehensive review delineates the multifaceted interactions between influenza viruses and ocular tissues. The research demonstrates that influenza affects various parts of the eye—from anterior to posterior segments—leading to a spectrum of complications ranging from mild conjunctivitis to severe vision-threatening conditions. Conjunctivitis remains the most common influenza-related ocular condition across all subtypes, although H7 avian influenza viruses exhibit the strongest ocular tropism. Although the public health benefits of influenza vaccination remain essential, medical professionals have documented sporadic adverse ocular events. The article emphasizes that ophthalmologists and healthcare providers must remain vigilant during influenza outbreaks to promptly detect and effectively manage these complications. Future research should aim to elucidate the precise pathophysiological mechanisms underlying influenza-associated ocular complications and to develop targeted therapeutic approaches.

## Figures and Tables

**Table 1:** Comparative analysis of clinical and epidemiological characteristics among influenza virus types.

Characteristics	Influenza A Virus (IAV)	Influenza B Virus (IBV)	Influenza C Virus (ICV)	Influenza D Virus (IDV)
**Host Range**	Humans, aquatic birds (natural reservoir), pigs and other mammals	Primarily humans	Humans, pigs, and dogs	Primarily cattle and swine
**Clinical Manifestations**	Acute onset of fever (38–41 °C), dry cough, headache, myalgia, arthralgia, ocular pain, photophobia, conjunctival congestion, sore throat, nasal congestion, fatigue	Similar to IAV, with fever, cough, and headache as predominant symptoms; biphasic fever pattern may occur in children	Mild upper respiratory inflammation with cough, low-grade fever, malaise, particularly common in children aged 1–6 years	Mild rhinitis and tracheitis in animals; human pathogenicity undetermined
**Complications**	Viral/bacterial pneumonia, bronchitis, sinusitis, otitis media, myocarditis, myositis, encephalopathy, respiratory failure, ARDS, septic shock	Similar to IAV; bacterial pneumonia and cardiac injury, notably common in pediatric fatal cases	Occasional bronchitis and pneumonia, primarily in children under 2 years	Severe symptoms in animals typically require co-infection with other respiratory pathogens
**Transmission**	Primarily via respiratory droplets (>5 μm) and aerosols (<5 μm); contact transmission possible; incubation period 1–4 days	Transmission patterns identical to IAV; similar incubation period	Limited human-to-human transmission	Animal transmission documented; human transmission patterns unknown
**Susceptible Population**	High-risk groups: children <5 years, elderly >65 years, pregnant women, immunocompromised individuals, those with chronic conditions	Similar to IAV, but potentially more severe in healthy children	Predominantly affects children aged 1–6 years	Primarily observed in animal populations
**Antigenic Variation**	Both antigenic drift and shift	Antigenic drift only	Slow antigenic evolution	Variation patterns not well characterized
**Vaccine Strategy**	Included in seasonal influenza vaccines (H1N1/H3N2 subtypes)	Included in seasonal vaccines (Victoria/Yamagata lineages)	Not included due to mild symptoms	No vaccine available
**Epidemiological Features**	Widespread globally; seasonal epidemics in temperate regions during winter; capable of causing pandemics (e.g., H1N1/H3N2); year-round circulation in tropical regions	Co-circulates with IAV; seasonal pattern similar to IAV; does not cause pandemics	Sporadic cases throughout the year; no epidemic potential	Limited to animal populations; no documented seasonal pattern in humans

**Table 2:** Summary of reported ocular manifestations associated with influenza virus infection.

Reference	Country/Regions	Study Design	Virus Subtype	Study Subjects	Ocular Symptoms	Other Symptoms/Complications	Transmission Route	Detection Method	Treatment and Prognosis
Taylor et al. (1977) [26]	UK	Case report	IAV (H7N7)	1 lab worker	Follicular conjunctivitis, keratitis with intraepithelial opacities	Mucopurulent discharge	Laboratory exposure	Viral culture	Self-limiting over 2–3 weeks
Webster et al. (1981) [27]	USA	Case series	IAV (H7N7)	4 marine biologists	Purulent conjunctivitis, periorbital swelling	Pain	Direct exposure to infected seals	Not attempted	Recovery in 4–5 days
Kurtz et al. (1996) [34]	UK	Case report	IAV (H7N7)	1 housewife	Unilateral conjunctivitis	None	Duck house exposure	Viral culture, ELISA	Recovery in 4 days
Puzelli et al. (2005) [28]	Italy	Serological survey	IAV (H7N1, H7N3)	983 poultry workers	Conjunctivitis	ILI	Occupational exposure	Serology	Not specified
Koopmans et al. (2004) [29]	Netherlands	Outbreak investigation	IAV (H7N7)	453 exposed persons	Conjunctivitis	ILI	Poultry exposure	RT-PCR	Oseltamivir prophylaxis
Tweed et al. (2004) [30]	Canada	Case report	IAV (H7N3)	2 poultry workers	Conjunctivitis	Mild ILI	Poultry exposure	Not specified	Not specified
Editorial team (2007) [31]	UK	Outbreak report	IAV (H7N2)	20 exposed persons	Conjunctivitis	ILI	Poultry exposure	Not specified	Antiviral treatment
Lopez-Martinez et al. (2013) [32]	Mexico	Case report	IAV (H7N3)	2 poultry workers	Conjunctivitis	Not reported	Poultry exposure	Genomic analysis	achieved complete recovery following targeted therapeutic intervention.
Puzelli et al. (2014) [33]	Italy	Case series	IAV (H7N7)	3 poultry workers	Conjunctivitis	Not reported	Poultry exposure	Genetic analysis	Not specified
Rothova et al. (2011) [66]	Netherlands	Case report	IAV (H1N1)	1 patient	Bilateral VZV-associated panuveitis	None	Vaccination	PCR, GWC	Valacyclovir, ganciclovir
Koul et al. (2013) [67]	India	Case report	IAV (H1N1)	1 adult	Subconjunctival hemorrhage	Rash, respiratory symptoms	Not specified	RT-PCR	Oseltamivir
Goyal et al. (2019) [64]	USA	Case series	IBV	2 pediatric patients	Mucositis, conjunctivitis	SJS	Not specified	Not specified	Antibiotics, steroids
Du Ry van Beest Holle et al. (2005) [63]	Netherlands	Cohort study	IAV (H7N7)	56 household contacts	Conjunctivitis	ILI	Human-to-human	Serology	Not specified
Lopez-Prats et al. (2010) [68]	Spain	Case report	IAV (H1N1)	45-year-old woman	Unilateral bleeding follicular conjunctivitis	Prior influenza-like illness	Human-to-human	PCR of conjunctival secretion	Symptomatic treatment with topical NSAIDs and washes associated with topical ganciclovir; resolved after 15 days
Shields et al. (2020) [69]	USA	Case report	IAV	1 pediatric patient	Macular neuroretinopathy	Cerebral involvement	Not specified	MRI, OCT	IV methylprednisolone
Yoshino et al. (2018) [70]	Japan	Case report	IAV	1 adult	Vogt-Koyanagi-Harada disease	Meningitis	Not specified	Antigen test	Peramivir, steroids
Lai et al. (2011) [71]	Taiwan	Case report	IAV	1 pediatric patient	Bilateral anterior uveitis, papillitis, neuroretinitis	Upper respiratory infection	Not specified	Not specified	Steroid pulse therapy
Gümbel et al. (2004) [72]	Germany	Case report	IAV	1 adult	Bilateral panuveitis	Upper respiratory infection	Not specified	Serology	Amantadine, immunoglobulins
Breker et al. (2015) [73]	USA	Case report	IAV (H1N1)	1 pediatric patient	Retinal and lateral geniculate nucleus infarction	Encephalopathy	Not specified	Not specified	IV steroids, IVIG, plasmapheresis
Nakagawa et al. (2017) [74]	Japan	Case report	IAV	1 adult	Bilateral anterior uveitis, unilateral optic neuritis	Upper respiratory infection	Not specified	PCR (aqueous)	Oseltamivir, steroids
Brydak-Godowska et al. (2019) [6]	Poland	Case series	IAV	4 adults	APMPPE, serous macular detachment	Upper respiratory infection	Not specified	RT-PCR, HAI	Systemic prednisone
Roesel et al. (2010) [75]	Germany	Case report	IAV (H1N1)	1 pediatric patient	Uveal effusion syndrome	Fever, cough	Not specified	PCR	Oral prednisolone, doxycycline

Abbreviations: APMPPE: acute posterior multifocal placoid pigment epitheliopathy; ELISA: enzyme-linked immunosorbent assay; GWC: Goldmann–Witmer coefficient; HAI: hemagglutination inhibition test; IAV: influenza A virus; IBV: influenza B virus; ILI: influenza-like illness; IVIG: intravenous immunoglobulin; MRI: magnetic resonance imaging.; NSAIDs: non-steroidal anti-inflammatory drugs; OCT: optical coherence tomography; PCR: polymerase chain reaction; RT-PCR: reverse transcription polymerase chain reaction; SJS: Stevens–Johnson syndrome; VZV: varicella zoster virus.
